# Childhood predictors of self-harm, externalised violence and transitioning to dual harm in a cohort of adolescents and young adults

**DOI:** 10.1017/S0033291723000557

**Published:** 2023-11

**Authors:** Sarah Steeg, Bushra Farooq, Peter Taylor, Matina Shafti, Becky Mars, Nav Kapur, Roger T Webb

**Affiliations:** 1 Division of Psychology and Mental Health, Centre for Mental Health and Safety, University of Manchester, Manchester, UK; 2 Manchester Academic Health Science Centre, University of Manchester, Manchester, UK; 3 Population Health Sciences, Bristol Medical School, University of Bristol, Bristol, UK; 4 Centre for Academic Mental Health, University of Bristol Medical School, Bristol, UK; 5 NIHR Greater Manchester Patient Safety Translational Research Centre, Manchester, UK; 6 Greater Manchester Mental Health NHS Foundation Trust, Manchester, UK

**Keywords:** ALSPAC, childhood adversity, dual harm, psychosocial risk factors, self-harm, violence

## Abstract

**Background:**

The aetiology of dual harm (co-occurring self-harm and violence towards others) is poorly understood because most studies have investigated self-harm and violence separately. We aimed to examine childhood risk factors for self-harm, violence, and dual harm, including the transition from engaging in single harm to dual harm.

**Methods:**

Data from the Avon Longitudinal Study of Parents and Children, a UK-based birth cohort study, were used to estimate prevalence of self-reported engagement in self-harm, violence, and dual harm at ages 16 and 22 years. Risk ratios were calculated to indicate associations across various self-reported childhood risk factors and risks of single and dual harm, including the transition from single harm at age 16 years to dual harm at age 22.

**Results:**

At age 16 years, 18.1% of the 4176 cohort members had harmed themselves, 21.1% had engaged in violence towards others and 3.7% reported dual harm. At age 22 the equivalent prevalence estimates increased to 24.2, 25.8 and 6.8%, respectively. Depression and other mental health difficulties, drug and alcohol use, witnessing self-harm and being a victim of, or witnessing, violence were associated with higher risks of transitioning from self-harm or violence at age 16 to dual harm by age 22.

**Conclusions:**

Prevalence of dual harm doubled from age 16 to 22 years, highlighting the importance of early identification and intervention during this high-risk period. Several childhood psychosocial risk factors associated specifically with dual harm at age 16 and with the transition to dual harm by age 22 have been identified.

## Introduction

‘Dual harm’ refers to the co-occurrence of self-harm and physical harm directed towards other people (Richmond-Rakerd et al., 2019). No single established definition of dual harm exists, though it is generally understood to involve a range of self-harming behaviours, with or without suicidal intent, alongside physical aggression towards another person with varying degrees of severity and of intent (Richmond-Rakerd et al., 2019; Shafti, Taylor, Forrester, & Pratt, 2021). Previous research on dual harm has mostly been conducted in clinical populations or has examined only those self-harm and interpersonal violence episodes that reach the attention of health services or criminal justice agencies. In general population settings, prevalence of dual harm is around 2–5% (Harford, Chen, Kerridge, & Grant, 2018; Harford, Yi, & Grant, 2013; Richmond-Rakerd et al., 2019). Prevalence of dual harm in prison settings has been reported as 11% (Slade, Forrester, & Baguley, 2020), and in clinical settings it varies from 13% among psychiatric outpatients (Scocco et al., 2019) to between 28 and 50% in inpatient settings (Boxer, 2010; Plutchik, Vanpraag, & Conte, 1989).

Several childhood risk factors are associated with adolescent self-harm, including bullying (Fisher et al., 2012), impulsivity, anxiety and depression and self-harm among peers (Madge et al., 2011), as well as parents experiencing mental illness, violence and self-harm (Pitkanen, Remes, Aaltonen, & Martikainen, 2019). Many of these childhood risk factors also heighten an individual's risk of being violent towards others during adolescence (Henry, Tolan, Gorman-Smith, & Schoeny, 2012; World Health Organization & WHO Collaborating Centre for Violence Prevention, 2010). In general population settings, where dual harm outcomes were identified from health services and criminal justice system records, dual harm risks were higher among people whose parents had experienced social adversity, including unemployment, substance misuse, self-harm and violence (Carr et al., 2020). In a Dutch community-based study, which was oversampled for emotional and behavioural symptoms, young people who had engaged in dual harm reported more substance misuse, parental hostility and more emotional and behavioural problems (Spaan et al., 2022). In summary, a number of adverse experiences and risk factors in childhood and adolescence have been found to be linked with self-harm and violence, with some also associated with increased risks of dual harm.

Interpersonal violence and self-harm also share several key adverse outcomes. For example, self-harm and violence are each associated with heightened risks of suicide and mental disorder (Bjorkenstam, Hjern, Bjorkenstam, & Kosidou, 2018; Borschmann et al., 2017; Goldman-Mellor et al., 2014; Harford et al., 2013; Hawton et al., 2020; Mars et al., 2014). While less is understood about the outcomes for people engaging in dual harm, some evidence has found them to be worse than those who engage in just one of the two types of harmful behaviour. For example, the presence of aggression has been found to increase lethality of suicide attempts (Gvion & Apter, 2011; Richmond-Rakerd et al., 2019). In addition, those who had reported dual harm in adolescence were more likely to continue to engage in self-harm or violence than those who had reported single harm (Steinhoff, Ribeaud, Eisner, & Shanahan, 2022). Risk of premature mortality from unnatural causes is also considerably higher among people with a history of dual harm compared to single harm (Steeg et al., 2019). There is a strong rationale, therefore, for understanding more about the progression to dual harm.

Identifying the specific risk factors associated with the co-occurrence of self-harm and violence is an important component of the evidence base for prevention and intervention for this especially risky group of young people. Evidence suggests that engaging in one harmful behaviour increases the likelihood of engaging in another (Goldman-Mellor et al., 2014; O'Donnell, House, & Waterman, 2015; Sahlin et al., 2017). Based on this shared aetiology, the imperative for investigating dual harm as a discrete entity is increasing (Mok et al., 2016, 2018; Shafti et al., 2021). Furthermore, emerging evidence suggests that experiencing a greater number of risk factors in childhood is associated with increased risks for subsequent dual harm *v.* single harm, compared to experiencing one or two (Carr et al., 2020). However, evidence concerning how early adverse experiences in general population settings are associated with self-harm, violent behaviour and dual harm in the community is only recently beginning to emerge (Richmond-Rakerd et al., 2019; Steinhoff, Bechtiger, Ribeaud, Eisner, & Shanahan, 2022).

Much of the research conducted to date on dual-harm has been cross-sectional in nature, focussing on a single point in time. Such studies are limited, however, in what they can tell us about the development or emergence of dual-harm. Whilst self-harm and violence towards others can occur concurrently, we anticipate that in most cases an individual first engages in either self-harm or aggression to others, and then later in life transitions to engage in the other behaviour as well. For example, qualitative research has indicated that those who engage in dual harm attribute specific functions and meanings to self-harm and violence respectively. Self-harm and violence are each influenced by a complex set of psychological processes and social contexts (Pickering, Blagden, & Slade, 2022), suggesting it is less likely that dual harm would begin with the co-occurrence of both behaviours at a single point in time. Longitudinal data provides an opportunity to investigate what factors predict this transition from single harm to dual harm, and may help elucidate how dual harm develops over time. The aim of this study was to investigate the prevalence of dual-harm in a large national sample of adolescents, and to examine the links between childhood risk factors and dual-harm in adolescence. Utilising data from the Avon Longitudinal Study of Parents and Children (ALSPAC), our specific objectives were to estimate:

- prevalence of self-reported history of self-harm, violence towards others, and engaging in both harmful behaviours (‘dual harm’) at ages 16 and 22 years.
- The relationship between risk factors and dual-harm, including examining if dual-harm risks increased with a cumulative increase in the number of antecedent risk factors, and whether this dose-response relationship varied between single and dual harm.
- psychosocial risk factors among adolescents who transitioned from single harm (self-harm or violence towards others) at age 16 to dual harm at age 22 years

We hypothesised that exposure to familial violence and self-harm in peers and family members would infer a particularly high risk of transitioning from single harm to dual harm (Hypothesis 1). We also hypothesised there would be a steeper dose-response relationship gradient with increasing numbers of risk factors experienced for dual harm than for single harm (Hypothesis 2) (Carr et al., 2020; Richmond-Rakerd et al., 2019).

## Methods

### Study design and sample

The ALSPAC is an ongoing transgenerational cohort study examining influences on health and development across the life course. The ALSPAC study originally recruited 14 541 pregnant women with expected dates of delivery between 1st April 1991 and 31st December 1992 in the former county of Avon in South West England. When the oldest children were approximately 7 years of age, an attempt was made to bolster the initial sample with eligible individuals who had opted not to join the study originally. The total sample size for analyses using any data collected after the age of seven is therefore 15 454 pregnancies, resulting in 15 589 foetuses. Of these 14 901 were alive at 1 year of age (Boyd et al., 2013; Fraser et al., 2013; Northstone et al., 2019).

Research clinics and self-report questionnaires were used to collect data at regular intervals. The study website contains details of all the data that is available through a fully searchable data dictionary and variable search tool: http://www.bristol.ac.uk/alspac/researchers/our-data/. Study data were collected and managed using REDCap (Research Electronic Data Capture), which is hosted at the University of Bristol (Harris et al., 2009). This is a secure, web-based software platform that supports data capture for research studies. The current study is based a subsample of young people who completed questionnaires relating to self-harm and violence between ages 16 (4176 participants) and 22 (4726) years.

### Outcome measures, exposure variables and covariates

#### Outcome measures

Responses to all questions that asked participants if they had harmed themselves or been violent towards others were combined to derive the self-harm, violence and dual harm outcome measures at ages 16 and 22 (online Supplementary Table S1). Young people were asked in clinic settings and self-report questionnaires about their experiences of engaging in self-harm and violence. For instance, young people were asked in a questionnaire at ages 16 and 20 years if they had ever ‘hurt themselves on purpose in any way (e.g. by taking an overdose of pills, or by cutting themselves)’. In relation to violence outcomes, young people were asked in a clinic setting at ages 15 and 17 if they had ‘hit/kicked/punched someone else on purpose with the intention of really hurting them at least once’ (online Supplementary Table S1). For the age 16 outcome, data were derived from questionnaires administered at ages 15 (violence) and 16 (self-harm). For the age 22 outcome, data were derived from questionnaires administered at ages 18 (self-harm; violence), 21 (self-harm; violence) and 22 (violence). Participants were coded as having engaged in self-harm or violence if a positive response to any of these measures was recorded. We only included participants with information on the presence or absence of self-harm and violence for each of the items used to derive the outcome measures at ages 16 and 22 (as listed in online Supplementary Table S1 in the supplementary material).

#### Exposure variables

Measures of childhood risk factors were selected based on existing evidence concerning risk factors for self-harm, violence and dual harm. These included depression (age 13), attitudes towards violence (age 13), being a victim of violence (various items reporting experiences before age 11 years and between 11 and 17 years), exposure to self-harm among peers (age 16) and family members (age 16), drug use (age 15) and alcohol misuse (age 15), callous-unemotional traits (mother reported, age 13), behavioural and emotional dysregulation (mother reported, age 13) and body image satisfaction (age 13) (online Supplementary Table S1). Depression was measured at age 13 using the moods and feelings questionnaire. This self-reported questionnaire is used for assessing symptoms of depression in the past two weeks, with possible scores ranging from 0–26; an established cut-off of 12 was used to identify participants experiencing depression, with a score of 12 or higher indicating the presence of clinically significant depression (Thabrew, Stasiak, Bavin, Frampton, & Merry, 2018). Behavioural and emotional dysregulation was measured at age 13 using the self-report Strengths and Difficulties Questionnaire (SDQ). The SDQ is a validated tool used to measure conduct problems, hyperactivity/inattention, peer problems, emotional symptoms and pro-social behaviour (Goodman, Meltzer, & Bailey, 1998). A score of 16 or higher on the SDQ has been identified as indicating a higher level of need. While we intended to use this threshold for the present study, very few participants scored 16+; therefore, we present results according to the mean score. The mean score was also presented for items assessing participants’ attitude to violence, number of alcoholic drinks typically consumed on a day when the young person drinks alcohol and the number of callous unemotional traits reported.

#### Covariates

In addition to unadjusted estimates, we examined associations between exposures and outcomes by the following potential confounders: gender and socioeconomic position (derived from maternal and paternal social class and parental income at age 8 – online Supplementary Table S1). ‘Low’ social class was defined as one or both parents in classes III (Manual) to V, with the lower of maternal and parental class used. ‘Low’ income was defined as below the median of parental income in the sample. Low socioeconomic position included instances where both social class and income were categorised as ‘low’. The study website contains details of all the data that are available through a fully searchable data dictionary and variable search tool (The Avon Longitudinal Study of Parents and Children (ALSPAC), 2022).

### Statistical analysis

The characteristics of missing data were explored and, where appropriate, multiple imputation using the chained equations approach was used to impute missing values for exposure variables and covariates (White, Royston, & Wood, 2011). There were small differences in those with complete data; they were more likely to be from a higher socioeconomic background and a higher proportion were female. 50 datasets were imputed, using exposure, outcome and auxiliary variables as predictors of missing data (online Supplementary Table S2). Results from complete case analyses are also presented as online Supplementary material. Prevalence percentages or mean scores were estimated for exposure variables within each outcome group (no harm, self-harm, violence and dual harm). Separate multinomial regression models were generated to examine relative risk ratios (RRRs) between the risk factors and the four-category outcome variable (dual harm; self-harm only; violence only; neither harm). Separate models were conducted for outcomes at ages 16 and 22 years. To examine the transition from single harm at age 16 to dual harm at age 22 (Hypothesis 1) we estimated risk ratios for dual harm at age 22 years within the group reporting self-harm only or violence only at age 16 (and who contributed follow-up information at age 22 years), using a modified Poisson regression approach (Zou, 2004). We also examined RRRs for each outcome category according to the number of adverse factors experienced (0–2, 3–4 and 5+) (Hypothesis 2). Analyses were performed using Stata SE Release Version 15.1 (StataCorp, 2019).

We followed STROBE (Strengthening the Reporting of Observational Studies in Epidemiology) guidelines (von Elm et al., 2008). The protocol for our study was approved by ALSPAC and was also pre-registered on open science framework (https://osf.io/3sgz9/). Ethical approval for the study was obtained from the ALSPAC Ethics and Law Committee and the Local Research Ethics Committees.

## Results

### Description of the study cohort (objective 1)

The study cohort consisted of 4176 participants who completed questions relating to self-harm and violence outcomes at age 16 years and 4726 who completed those questions at age 22 (online Supplementary Table S1). In the overall study sample at age 16 (*N* = 4176), over half of the sample was female (56.5%), almost a quarter (23.1%) were from a low socioeconomic background, and almost a half (48.1%) of the study children's mothers were aged between 20 and 29 years at delivery. Ninety five per cent of children were from a white ethnic background. Characteristics of the sample at age 22 were similar. At age 16 years, 755/4176 (18.1%) were identified as having harmed themselves, 881 (21.1%) had engaged in violence towards others and 154 (3.7%) had engaged in dual harm. At age 22 the equivalent values increased to 1142 (24.2%), 1217 (25.8%) and 321 (6.8%), respectively.

### Prevalence of psychosocial risk factors in young people engaging in neither type of harm, self-harm, violence towards others and dual harm at age 16 and 22 years (objective 2)

Young people in the self-harm group were more likely to be female, while the group reporting acting violently towards others were more likely to be male (Tables 1 and 2). Lower socioeconomic position was more prevalent in both of the ‘single harm’ groups compared to the ‘neither harm’ group. At age 16, depression, being hit by friends or by someone in family before age 11, not being happy with body image, number of alcoholic drinks, drug use in past year and mean scores on the SDQ and questions relating to callous unemotional traits were more prevalent among people reporting dual harm than either of the single harm behaviours (Table 1). At age 22, being victim of dating violence, being hit with something by family member, being hit by family between 11 and 17 years, witnessing parental violence and having a family member who had self-harmed were additional factors that were more prevalent among those reporting dual harm compared to self-harm or violence alone (Table 2). The prevalence of risk factors in the single and dual harm groups at age 16 and 22, when using complete data only, were similar (online Supplementary Tables S3 and S4).

**Table 1.** Description of cohort: prevalence of exposure by dual harm status at age 16 years (pooled proportions from imputed data)*

Characteristic/exposure	Neither self-harm nor violence		Self-harm only		Violence only		Dual harm	
Categorical measures	*n*	%	*n*	%	*n*	%	*n*	%
Total	2386	57.1	755	18.1	881	21.1	154	3.7
Male	1392	58.3	132	17.5	647	73.4	106	68.8
Female	994	41.7	623	82.5	234	26.6	48	31.2
Low socioeconomic position	477	20.1	207	26.5	279	31.4	43	28.8
Depression (Short Mood and Feelings Questionnaire) score: Yes ⩾12 (age 13)	136	5.5	139	18.3	74	8.6	36	22.9
Victim of dating violence (age 13)	178	8.2	83	12.5	154	17.4	17	14.7
Hit by friends (age 12)	197	8.1	105	13.4	165	18.4	35	23.6
Hit by someone outside family before age 11^a^	25	1.1	21	2.4	35	3.3	9	4.2
Hit by someone in family before age 11^a^	106	4.7	77	11.1	98	11.9	25	15.7
Hit with something by family before age 11^a^	104	4.5	85	11.2	105	12.6	22	12.0
Hit by someone in family between age 11 and 17^a^	79	3.6	73	10.1	80	9.0	15	9.2
Witnessed parental violence ever (reported at age 21)	85	3.8	76	8.8	75	8.0	6	7.4
Family member self-harmed (age 16)	136	5.6	174	23.1	77	8.7	33	21.5
Close friend self-harmed (age 16)	734	30.9	585	77.2	286	31.9	121	78.6
Happy with body image (age 13)	1706	71.4	387	51.2	638	71.1	69	45.0
Drug use in past year (age 15)	40	1.8	43	6.1	80	9.0	24	15.9
Continuous measures	*n*	Mean	*n*	Mean	*n*	Mean	*n*	Mean
Number of drinks when typically drinks alcohol (age 15)	2386	1.5	755	2.1	881	2.5	154	2.9
Score on 13-item beliefs about violence scale (age 13)	2386	1.9	755	2.1	881	3.1	154	2.5
Strengths and Difficulties Questionnaire (mean score) (age 13)	2386	5.8	755	7.1	881	7.6	154	8.8
Callous unemotional traits (age 13)	2386	4.2	755	5.2	881	5.3	154	5.9

* Based on the cohort of individuals cases with data for self-harm/violence/dual harm at age 16 (*n* = 4176) and excluding individuals with missing data for sex (*n* = 5), length of pregnancy (*n* = 249), mother's age at delivery (*n* = 0), pregnancy size (singleton *v.* multiple) (*n* = 0).

a Reported at age 22.

**Table 2.** Description of cohort: prevalence of exposure by dual harm status at age 22 years (pooled proportions from imputed data)*

Characteristic/ exposure	Neither self-harm or violence		Self-harm only		Violence only		Dual harm	
Categorical measures	*n*	%	*n*	%	*n*	%	*n*	%
Total	2046	43.3	1142	24.2	1217	25.8	321	6.8
Female	1196	58.5	895	78.4	364	29.9	219	68.2
Male	850	41.5	247	21.6	853	70.1	102	31.8
Low socioeconomic position	407	20.0	298	25.2	376	31.0	93	29.2
Depression (Short Mood and Feelings Questionnaire) score: Yes ⩾12 (age 13)	111	5.3	180	15.3	92	7.8	75	22.9
Victim of dating violence (age 13)	147	7.7	115	11.5	185	15.7	53	18.6
Hit by friends (age 12)	165	8.0	150	12.5	207	16.9	74	24.1
Hit by someone outside family before age 11^a^	22	1.1	34	2.1	47	3.3	12	3.7
Hit by someone in family before age 11^a^	77	4.1	114	9.8	137	11.2	53	18.2
Hit with something by family before age 11^a^	75	4.0	114	9.7	150	12.3	47	15.2
Hit by someone in family between age 11 and 17^a^	53	3.0	96	8.3	117	9.3	41	13.5
Witnessed parental violence ever (reported at age 21)	67	3.4	97	7.4	95	7.7	36	12.5
Family member self-harmed (age 16)	106	5.1	203	18.4	108	8.8	70	21.9
Close friend self-harmed (age 16)	609	29.9	753	65.2	396	31.9	220	70.1
Happy with body image (age 13)	1475	72.1	618	54.6	881	71.6	157	48.6
Drug use in past year (age 15)	31	1.6	55	5.3	91	7.2	47	14.6
Continuous measures	*n*	Mean	*n*	Mean	*n*	Mean	*n*	Mean
Score on 13-item beliefs about violence scale (age 13)	2046	1.9	1142	2.0	1217	2.9	321	2.8
Mean number of drinks when typically drinks alcohol (age 15)	2046	1.5	1142	2.0	1217	2.3	321	2.8
Strengths and Difficulties Questionnaire (mean score) (age 13)	2046	5.7	1142	6.9	1217	7.4	321	8.5
Callous unemotional traits (age 13)	2046	4.1	1142	5.1	1217	5.2	321	5.9

* Based on the cohort of individuals cases with data for self-harm/violence/dual harm at age 22 (*n* = 4726) and excluding individuals with missing data for sex (*n* = 5), length of pregnancy (*n* = 249), mother's age at delivery (*n* = 0), pregnancy size (singleton *v.* multiple) (*n* = 0).

a Reported at age 22.

### Adjusted relative risks for self-harm, violence towards others and dual harm at age 16 and 22 years by presence of psychosocial risk factors (objective 2)

All of the exposures that we examined were associated with self-harm only and violence only (i.e. single harm) at age 16 and 22 (Table 3). At age 16, particularly high relative risks for self-harm were observed for young people experiencing depression (RR 3.29, 95% CI 2.51–4.31), a family member (4.29, 3.34–5.50) or close friend (6.44, 5.29–7.84) who had harmed themselves and drug misuse (3.60, 2.19–5.91). For violence at age 16, risks were particularly elevated for young people experiencing violence within the family (e.g. being hit with something by someone in their family: 3.00, 2.07–4.35) and misusing drugs (5.63, 3.67–8.63).

**Table 3.** Relative risk ratios (RRR) for the association between exposures and dual harm status at age 16 and 22 years: adjusted for sex and socioeconomic position (pooled proportions from imputed data)

Characteristic/exposure	Age 16 years				Age 22 years			
	Neither self-harm or violence (*N* = 2386)	Self-harm only (*N* = 755)	Violence only (*N* = 881)	Dual harm (*N* = 154)	Neither self-harm or violence (*N* = 2046)	Self-harm only (*N* = 1142)	Violence only (*N* = 1217)	Dual harm (*N* = 321)
	RRR	RRR (95% CI)	RRR (95% CI)	RRR (95% CI)	RRR	RRR (95% CI)	RRR (95% CI)	RRR (95% CI)
Depression (Short Mood and Feelings Questionnaire) score ⩾12 (age 13)	1 (ref)	3.29 (2.51–4.31)	2.08 (1.50–2.89)	4.82 (3.13–7.42)	1 (ref)	2.84 (2.18–3.72)	1.86 (1.35–2.57)	5.03 (3.58–7.07)
Mean attitude to violence score (age 13)	1 (ref)	1.15 (1.08–1.21)	1.37 (1.30–1.44)	1.26 (1.14–1.39)	1 (ref)	1.09 (1.03–1.14)	1.31 (1.25–1.37)	1.37 (1.28–1.47)
Victim of dating violence (age 13)	1 (ref)	1.73 (1.25–2.40)	1.98 (1.48–2.65)	1.95 (1.16–3.29)	1 (ref)	1.65 (1.22–2.22)	1.93 (1.46–2.55)	2.76 (1.90–4.02)
Hit by friends (age 12)	1 (ref)	2.32 (1.73–3.12)	1.98 (1.56–2.52)	4.05 (2.65–6.18)	1 (ref)	2.07 (1.59–2.71)	1.87 (1.48–2.37)	4.21 (3.04–5.83)
Hit by someone outside family before age 11^a^	1 (ref)	2.56 (1.10–5.96)	2.28 (1.04–5.00)	4.01 (1.34–11.98)	1 (ref)	2.17 (1.01–4.66)	2.53 (1.26–5.09)	3.48 (1.32–9.22)
Hit by someone in family before age 11^a^	1 (ref)	2.47 (1.71–3.56)	2.76 (1.94–3.91)	3.67 (2.05–6.57)	1 (ref)	2.45 (1.67–3.60)	2.97 (2.06–4.29)	5.03 (3.25–7.80)
Hit with something by family before age 11^a^	1 (ref)	2.72 (1.85–3.99)	3.00 (2.07–4.35)	2.87 (1.49–5.52)	1 (ref)	2.64 (1.79–3.89)	3.40 (2.34–4.94)	4.35 (2.72–6.96)
Hit by someone in family between age 11 and 17^a^	1 (ref)	2.86 (1.92–4.24)	2.85 (1.88–4.31)	2.58 (1.15–5.76)	1 (ref)	2.77 (1.82–4.19)	3.51 (2.31–5.32)	4.82 (2.91–7.97)
Witnessed parental violence ever (age 21)	1 (ref)	2.15 (1.44–3.23)	2.39 (1.53–3.72)	1.78 (0.71–4.59)	1 (ref)	2.05 (1.31–3.19)	2.53 (1.65–3.88)	3.72 (2.25–6.15)
Family member self-harmed (age 16)	1 (ref)	4.29 (3.34–5.50)	1.96 (1.37–2.81)	4.22 (2.74–6.48)	1 (ref)	3.62 (2.78–4.71)	2.16 (1.55–3.01)	4.76 (3.35–6.76)
Close friend self-harmed (age 16)	1 (ref)	6.44 (5.29–7.84)	1.52 (1.24–1.87)	8.36 (5.57–12.55)	1 (ref)	3.83 (3.24–4.52)	1.54 (1.28–1.86)	5.52 (4.15–7.34)
Happy with body image (age 13)	1 (ref)	0.50 (0.42–0.61)	0.78 (0.64–0.96)	0.35 (0.24–0.50)	1 (ref)	0.54 (0.45–0.64)	0.79 (0.66–0.95)	0.39 (0.30–0.51)
Drug use in past year (age 15)	1 (ref)	3.60 (2.19–5.91)	5.63 (3.67–8.63)	10.48 (6.01–18.26)	1 (ref)	3.37 (2.07–5.47)	4.59 (2.94–7.18)	10.15 (6.19–16.65)
Mean number of drinks when typically drinks alcohol (age 15)	1 (ref)	1.16 (1.10–1.21)	1.32 (1.26–1.38)	1.40 (1.29–1.51)	1 (ref)	1.15 (1.10–1.20)	1.29 (1.24–1.35)	1.39 (1.31–1.47)
Strengths and Difficulties Questionnaire (mean score) (age 13)	1 (ref)	1.07 (1.05–1.09)	1.07 (1.05–1.08)	1.13 (1.09–1.16)	1 (ref)	1.06 (1.04–1.08)	1.07 (1.05–1.09)	1.13 (1.10–1.15)
Callous unemotional traits (age 13)	1 (ref)	1.11 (1.08–1.14)	1.13 (1.09–1.16)	1.18 (1.12–1.24)	1 (ref)	1.10 (1.07–1.13)	1.13 (1.10–1.16)	1.19 (1.15–1.23)

a Reported at age 22.

At age 16, relative risks of dual harm compared to each of the single harm categories were particularly increased among young people reporting depression (RR 4.82, 95% CI 3.13–7.42), being hit by a friend (4.05, 2.65–6.18), having a close friend who had harmed themselves (8.36, 5.57–12.55), having drinking higher levels of alcohol (1.40, 1.29–1.51), drug misuse (10.48, 6.01–18.26), higher scores on the SDQ (1.13, 1.09–1.16) and higher levels of callous unemotional traits (1.18, 1.12–1.24), and were lower among individuals who were happy with their body image (0.35, 0.24–0.50) (Table 3). Similar associations were observed for risk of dual harm at age 22 (Table 3).

### Unadjusted relative risks for self-harm, violence towards others and dual harm at age 16 and 22 years by presence of psychosocial risk factors (objective 2)

Factors associated with self-harm, violence towards others and dual harm at ages 16 and 22 years were broadly similar in unadjusted analyses, though the relative risk estimates were lower in general (online Supplementary Table S5).

### Factors associated with the transition from self-harm or violence only at age 16 to dual harm by age 22 years (objective 3 and hypothesis 1)

Young people reporting self-harm or violence (but not dual harm) at age 16 had increased risks of dual harm by age 22 if they had experienced symptoms of depression (RR 3.33, 95% CI 2.34–4.74), dating violence (2.81, 1.91–4.11), endorsed proviolence attitudes (2.52, 1.86–3.42), were hit by friends (3.52, 2.48–5.01), had been a victim of violence within the family before age 11 (3.41, 2.28–5.10), or between the ages of 11 and 17 (3.21, 2.10–4.91), had been hit with an object by a family member (2.81, 1.83–4.33), witnessed parental violence (3.42, 2.26–5.18), had a family member (2.63, 1.81–3.82) or close friend (5.74, 3.87–8.51) harm themselves, reported drug use in the past 12 months (3.56, 2.27–5.58), consumed higher levels of alcohol (2.92, 2.11–4.03), had higher SDQ scores (2.90, 1.57–5.35), and callous unemotional traits (3.04, 2.21–4.17) (Table 4).

**Table 4.** Risk ratios (RRs) indicating predictors of transition from single harm at age 16 years to dual harm at age 22 years: adjusted for sex and socioeconomic position (pooled estimates from imputed data)

Characteristic/exposure	Dual harm at age 22 years		Risk ratio	95% CI
	*n*	%		
All persons who reported single harm at age 16 (*N* = 1636)	167	10.2		
Depression (Short Mood and Feelings Questionnaire; score ⩾12) (age 13)	39	23.4	3.33	2.34–4.74
Attitude to violence score (age 13)	93	55.7	2.52	1.86–3.42
Victim of dating violence (age 13)	36	21.6	2.81	1.91–4.11
Hit by friends (age 12)	39	23.4	3.52	2.48–5.01
Hit by someone outside family before age 11^a^	–	–	–	–
Hit by someone in family before age 11^a^	28	16.8	3.41	2.28–5.10
Hit with something by family before age 11^a^	25	15.0	2.81	1.83–4.33
Hit by someone in family between age 11 and 17^a^	26	15.6	3.21	2.10–4.91
Witnessed parental violence ever (age 21)	30	18.0	3.42	2.26–5.18
Family member self-harmed (age 16)	37	22.2	2.63	1.81–3.82
Close friend self-harmed (age 16)	99	59.3	5.74	3.87–8.51
Happy with body image (age 13)	88	52.7	0.87	0.64–1.18
Drug use in past year (age 15)	23	13.8	3.56	2.27–5.58
Higher levels of alcohol (age 15)	101	60.5	2.92	2.11–4.03
Strengths and Difficulties Questionnaire score (age 13)	11	6.6	2.90	1.57–5.35
Callous unemotional traits (age 13)	106	63.5	3.04	2.21–4.17

a Reported at age 22.

–Cell count contains 5 or fewer participants so not reported.

### Relative risks of self-harm, violence and dual harm by number of psychosocial risk factors (objective 2 and hypothesis 2)

Risks of both self-harm and violence increased incrementally as the number of risk factors experienced by the young people increased (Table 5). However, the risks of dual harm increased by a far greater degree; around three to four times more than that of the values observed for the single harm groups. The pattern of results was consistent at ages 16 and 22.

**Table 5.** Relative risk ratios (RRR) for the association between exposure to total number of childhood risk factors and dual harm status at age 16 and 22 years: adjusted for sex and socioeconomic position (pooled estimates from imputed data)

Total number of exposures	Neither self-harm or violence		Self-harm only		Violence only		Dual harm	
Age 16 years	*N* = 2386	RRR (95% CI)	*N* = 755	RRR (95% CI)	*N* = 881	RRR (95% CI)	*N* = 154	RRR (95% CI)
0–2	1397	1 (ref)	156	1 (ref)	274	1 (ref)	20	1 (ref)
3–4	749	1 (ref)	313	3.51 (2.75–4.47)	344	2.37 (1.89–2.97)	47	5.27 (2.80–9.90)
5+	240	1 (ref)	286	10.79 (8.21–14.19)	263	5.91 (4.57–7.64)	87	28.99 (16.07–52.30)
Age 22 years	*N* = 2046	RRR (95% CI)	*N* = 1142	RRR (95% CI)	*N* = 1217	RRR (95% CI)	*N* = 321	RRR (95% CI)
0–2	1226	1 (ref)	319	1 (ref)	400	1 (ref)	52	1 (ref)
3–4	632	1 (ref)	453	2.76 (2.24–3.39)	470	2.25 (1.81–2.80)	103	4.36 (2.84–6.72)
5+	188	1 (ref)	370	7.39 (5.76–9.47)	347	5.97 (4.65–7.67)	166	23.94 (15.74–36.40)

## Discussion

### Main findings

Around one in fifteen young people had engaged in dual harm by age 22 years, almost double that of the prevalence found at age 16 years. Compared to the single harm behaviours, dual harm at age 16 was associated with higher levels of mental health difficulties, self-harm and violence among friends and alcohol and drug misuse. We hypothesised that experiencing self-harm or violence among family and peers would be associated with particularly elevated risks of transitioning to dual harm. We found that reporting depression, being hit by a friend or by another person outside of their family, having a family member harm themselves, reporting drinking higher levels of alcohol, drug misuse, higher SDQ scores and higher levels of callous unemotional traits were all associated with two- to three-fold increases in risk of transitioning from either of the single harm (self-harm/violence) outcomes at age 16 to dual harm by age 22. However, an especially high risk of transitioning to dual harm was observed among people who had a close friend that had self-harmed. While risks of both self-harm and externalised violence increased incrementally as the number of risk factors experienced by the young people increased, the equivalent risk gradient for dual harm was considerably steeper, in line with our second hypothesis.

### Comparison with existing literature

In our study, higher levels of drug and alcohol misuse and being a victim of, or witnessing, violence were linked to higher risks of dual harm at age 16 and 22 years. In a study of Danish young people aged 15–35 years, exposure to parental violence and substance misuse and being a victim of interpersonal violence were also found to be strong predictors of dual harm resulting in contact with health or criminal justice services (Carr et al., 2020). Previous research conducted in the UK on dual harm in the community also found a strong link between childhood maltreatment and elevated dual harm risk subsequently (Richmond-Rakerd et al., 2019). The same study also found that young people engaging in dual harm had rates of contact with mental health services that were no higher than those experiencing single harm, despite their higher rates of childhood adversity and poorer mental health (Richmond-Rakerd et al., 2019). Early intervention among young people experiencing multiple adversities, including among those experiencing single harm in adolescence, should address the potential for escalation to dual harm, especially considering their poorer prognosis in terms of continuing adversity and heightened risk of dying at a young age (Harford et al., 2013; Steeg et al., 2019).

While previous research has focused on the transition from adolescent self-harm to dual harm (Richmond-Rakerd et al., 2019), we examined childhood risk factors associated with moving from either of the single harm measures (self-harm or externalised violence) at age 16 years to dual harm at age 22. We found that exposure to violence or self-harm among close peers and family members, experiencing depression and other mental health difficulties and higher levels of drug and alcohol use were associated with the transition from single harm age 16 to dual harm by age 22. Given our finding that the prevalence of dual harm doubled between the ages of 16 and 22 years, these experiences are likely to be important markers of heightened risk, highlighting the possible routes to dual harm among young people. Recognising which specific adverse experiences put young people most at risk of dual harm is an important step in understanding treatment needs among young people engaging in either self-harm or interpersonal violence.

Our findings that young people had particularly high risks of transitioning to dual harm if they reported higher levels of drug misuse and alcohol use, higher SDQ scores, callous unemotional traits (potential markers for antisocial behaviour and psychopathy), depression and witnessing interpersonal violence or self-harm among peers or family extend existing evidence. Drug misuse, being exposed to peer and family violence and self-harm and emotional dysregulation have previously been identified as risk factors associated with dual harm (Carr et al., 2020; Richmond-Rakerd et al., 2019). We provide further evidence of risk factors for dual harm, in cases where one of the single harm behaviours had already been identified. Shafti et al., (Shafti et al., 2021), in their cognitive-emotional model of dual harm, suggest that psychological drivers including emotional regulation and interpersonal motivations (e.g. communication of distress) are characteristic of dual harm. Impulsivity and secondary psychopathy [in response to environmental adversity (Sethi et al., 2018)] were also proposed as having an aetiological role in dual harm, findings that are supported by what our study has revealed regarding callous unemotional traits. Along with our findings relating to cumulative number of risk factors experienced, the evidence provides further rationale for targeting interventions to young people experiencing violence and self-harm among family and peers as well as psychological processes such as secondary psychopathy, emotional dysregulation, and impulsivity, particularly where a young person has already engaged in self-harm or violence.

In contrast to our findings, previous research, in both studies of dual harm in non-clinical settings (Richmond-Rakerd et al., 2019) and in a clinical population (Plutchik et al., 1989; Richmond-Rakerd et al., 2019), has not identified higher risks of depression among people engaging in dual harm compared to single harm. One study of psychiatric outpatients with severe mental disorder (Scocco et al., 2019) reported higher depression scores among the dual harm group compared to the group engaging solely in violence towards others (though not compared to the self-harm only group). However, we found a greater risk of dual harm than both single harm outcomes among adolescents reporting clinically significant levels of depression at age 13 years, a novel finding that merits further investigation.

### Implications

In our study, exposure to peer and family self-harm or externalised violence was associated with dual harm at 16 and the transition from single harm at age 16 to dual harm by age 22 years. In relation to violence prevention, WHO recommends family-based interventions targeting childhood maltreatment by parents and caregivers, further recommending that violence prevention strategies also take into account societal inequalities that contribute to greater violence risk (World Health Organization & WHO Collaborating Centre for Violence Prevention, 2010). Evidence for school-based interventions for preventing violence among adolescents is limited but suggests that whole school-based programmes may offer some benefit, and that targeted interventions may be more effective than universal ones (Cox et al., 2016; Gavine, Donnelly, & Williams, 2016; Kovalenko, Abraham, Graham-Rowe, Levine, & O'Dwyer, 2020). Our findings suggest that targeting interventions towards young people experiencing depression and alcohol and substance misuse could help reduce the prevalence of dual harm. In terms of effective interventions for treating adolescents who have engaged in self-harm, the evidence is relatively weak, although therapeutic assessment, mentalisation-based therapy and dialectical behaviour therapy may improve treatment adherence and reduce risk of self-harm repetition risk (Witt et al., 2021). Focussing on adolescents experiencing depression and alcohol or substance misuse may help to prevent the transition to dual harm, a group of individuals who are at even greater risk. In one study of young people who had attended hospital following self-harm, most problems reported by adolescents were family- and school-related, suggesting interventions should be embedded in community and educational settings as well as health services (Townsend et al., 2022). Indeed, the majority of adolescents who harm themselves do not present to health services (Geulayov et al., 2018). A recent study suggested positive student-teacher experiences were linked to the cessation of single harm among adolescents (Steinhoff et al., 2022b). Our study's findings relating to higher risks of dual harm among young people witnessing violence and self-harm among peers and family contribute to evidence that addressing exposure to violence and self-harm within family and school settings is likely to be a vital component of effective interventions.

There is currently no clinical guidance for treating young people who engage in dual harm, although theoretical frameworks for understanding and managing dual harm are beginning to emerge (Pickering et al., 2022; Shafti et al., 2021). Cross-cutting interventions addressing self-harm and different types of interpersonal violence are potentially beneficial, but require further evaluation (Decker, Wilcox, Holliday, & Webster, 2018; Lubell & Vetter, 2006). To date, there is very little evidence regarding interventions focussing on both self-harm and violence. However, there is some consensus that such interventions should target individual-level factors such as improving skills in interpersonal problem-solving for young people at high risk of self-harm and violent behaviours. They should also focus on school-level factors, with recommended approaches including enhancing the quality of relationships with peers and staff and improving the overall school environment (e.g. reducing levels of bullying) (Lubell & Vetter, 2006). Optimising the potential effectiveness of interventions would require collaborative working across educational, criminal justice, mental health, primary care and emergency medicine settings. Involving young people in the design and implementation of interventions has also been identified as an important component of improving their acceptability and efficacy in preventing self-harm and interpersonal violence (Edwards, Jones, Mitchell, Hagler, & Roberts, 2016; Witt et al., 2021).

### Strengths and limitations

Examination of a general population cohort was a key strength of this study because most young people who have self-harmed do not present to clinical services (Hawton, Rodham, Evans, & Weatherall, 2002). Using ALSPAC data enabled us to examine self-harm and externalised violence in a community sample of young people in the UK. However, some limitations regarding the generalisability of the ALSPAC cohort should be noted; young people enrolled in the cohort had a higher level of educational attainment at age 16 compared to a national comparison sample and were less likely to be eligible for free school meals (Boyd et al., 2013). These differences may limit the generalisability of our findings. Specifically, given that we found higher prevalence of single and dual harm among young people of lower socioeconomic position, our findings may result in underestimation of the prevalence of these outcomes. Furthermore, a larger proportion of participants in the ALSPAC sample are of White ethnicity (96.1%) than in the national population (86.5%) (Boyd et al., 2013). In our cohort, a higher proportion with complete data were found to be from a higher socioeconomic background and were female, as previously found in ALSPAC data (Howe, Tilling, Galobardes, & Lawlor, 2013). These differences may result in underestimation of the strength of the relationship between socioeconomic inequalities and the outcomes that we examined. However, the differences between complete and multiply-imputed data in our study were small.

To ensure capture of self-harm and violence episodes for as many participants as possible, these outcome data were derived from measures collected at several time points, although a degree of misclassification may have thereby arisen; for example, if the self-harm or violence occurred after the participant completed the relevant study questions. In addition, some exposures were measured relatively close in time to the outcome measures while others were considered over a broader time period (e.g. retrospectively at ages 11–17 years). This may have affected the accuracy of participants’ self-reporting of exposure measures. The use of more refined measures for some of the study variables, taking into account the frequency and intensity of participants’ experiences, for instance, could lead to a greater level of insight into the associations observed between exposures and outcomes. We did not adjust for multiple testing due to our study being exploratory rather than confirmatory in nature, and to avoid overly conservative correction of *p* values (Bender & Lange, 2001). However, this means there is a higher probability of reporting false positive findings among the associations that we have reported. We were able to examine a broad range of childhood experiences, recorded as part of a longitudinal study conducted over many years. Although we cannot infer causal relationships between the childhood factors examined and the subsequent single and dual harm outcomes, our findings provide important contextual information. For example, understanding the risk factors that are associated with dual harm could be used in the design of appropriate interventions as well as help identification of individuals who are most at risk of engaging in dual harm.

## Conclusions

The prevalence of dual harm in this general population cohort doubled from age 16 to 22 years, highlighting the importance of early identification of and intervention for this high-risk group. Frequent opportunities to intervene are likely to arise as these individuals often encounter health, social care, and criminal justice services. We identified several childhood experiences associated specifically with dual harm at age 16 and with the transition to dual harm by age 22. Findings provide evidence supporting emerging novel models for dual harm and could help inform the development of interventions.

## Supporting information

Steeg et al. supplementary material Steeg et al. supplementary material

## Supplementary material

The supplementary material for this article can be found at https://doi.org/10.1017/S0033291723000557.
