# Supplementary material for: Childhood predictors of self-harm, externalised violence and transitioning to dual harm in a cohort of adolescents and young adults
Source: Psychol Med. 2023 Mar 31;53(15):7116–26. doi: 10.1017/S0033291723000557 (PMC10719627; doi:10.1017/S0033291723000557)
Supplement: Steeg et al. supplementary material [file S0033291723000557sup001.docx]

**Table S1: Derivation of exposure, outcome, and confounding variables**

| **Variable** | **Description** | **Time period covered** | **Variable type** | **Variable recoded** | **Age of child when measured** | **Source** | **Variable name** |
| --- | --- | --- | --- | --- | --- | --- | --- |
| *Outcome variables* |  |  |  |  |  |  |  |
| Dual harm at age 16 | ‘Hurt themselves on purpose in any way (e.g. by taking an overdose of pills, or by cutting themselves)’ | Lifetime | Binary: Yes/No | N/A | 16 | Child-completed questionnaire | ccs6530 |
| Dual harm at age 16 | ‘Frequency YP has hit/kicked/punched someone else on purpose with the intention of really hurting them at least once’ | Past year | Not at all  Once  2-5 times  6+ times | Yes = At least once  No = Not at all | 15.5 | Child clinic-based questionnaire | fh8313 |
| Dual harm at age 22 | ‘Self-harm indicator’ | Past year | Binary: Yes/No | N/A | 17.5 | Child clinic-based questionnaire | FJCI369 |
| Dual harm at age 22 | ‘Hurt themselves on purpose in any way (e.g. by taking an overdose of pills, or by cutting themselves)’ | Lifetime | Binary: Yes/No | N/A | 20+ | Child-completed questionnaire’ | CCU2040 |
| Dual harm at age 22 | ‘During last year YP hit, kicked, punched or attacked someone with the intention of really hurting them’ | Past year | Binary: Yes/No | N/A | 17.5 | Child clinic-based questionnaire | FJAA1150 |
| Dual harm at age 22 | ‘During last year YP hit, kicked, punched or attacked someone with the intention of really hurting them’ | Past year | Not at all  Once  2-5 times  6+ times | Yes = At least once  No = Not at all | 18 | Child-completed questionnaire | CCT6007 |
| Dual harm at age 22 | ‘In past year, frequency: hit, kicked or punched someone else on purpose with the intention of really hurting them’ | Past year | Not at all  Once  2-5 times  6+ times | Yes = At least once  No = Not at all | 20+ | Child-completed questionnaire | CCU3507 |
| Dual harm at age 22 | In past year, frequency: hit, kicked or punched someone else on purpose with the intention of really hurting them’ | Past year | Not at all  Once  2-5 times  6+ times | Yes = At least once  No = Not at all | 22+ | Child-completed questionnaire | YPB4487 |
| *Confounding variables* |  |  |  |  |  |  |  |
| Socio-economic position | Maternal and paternal social class | At time of questionnaire | I  II  III (non-manual)  III (manual)  IV  V  Armed forces | Low = III (Manual) to V | Birth | Mother-completed questionnaire | c755 – maternal social class  c765 – paternal social class |
| Socio-economic position | Parental income | At time of questionnaire | <£100  £100 - £199  £200 - £299  £300 - £399  £400+ | Below the median of parental income in the sample | 8 | Mother-completed questionnaire | n8130 - average family take-home income per week |
| *Exposure variables* |  |  |  |  |  |  |  |
| Depression | Total score on 13 item scale on moods and feelings questionnaire | Past two weeks | Scale derived from categorical items: True (2)  Sometimes (1)  Not at all (0) | Score >=12 = yes) | 13.5 | Child – clinic based | fg7226 |
| Attitude to violence | Total score on 13 item scale on beliefs about violence | N/A | Scale derived from categorical items:  Strongly agree/agree (2)  Neither agree/disagree (1)  Disagree/strongly disagree (0) | Mean score | 13.5 | Child – clinic based | fg4560-4572 |
| Exposure to violence | Victim of dating violence - any one or more of the following:   - Someone they have been out with has: intentionally scratched teenager - intentionally slapped teenager - intentionally kicked teenager - intentionally bent fingers of teenager - intentionally pushed/grabbed/shoved teenager - intentionally thrown something at teenager - intentionally hit with their fist teenager   Another form of violence has been used against teenager in a romantic relationship | Lifetime | Binary response:  Yes/No | N/A | 13.5 | Child – clinic based | fg4420-4437 |
|  | Hit/beaten up by friends and peers | Lifetime | Binary: Yes/No | N/A | 12.5 | Child – clinic based | ff6020 |
|  | Adult outside the family actually kicked, punched, hit respondent with something that could hurt respondent or physically attacked respondent in another way | Lifetime before age 11 | Categorical response:  Never/Rarely/Sometimes/Often/Very often | Yes = Rarely/  Sometimes/  Often/  Very often  No = Never | Before age of 11 (retrospective reporting at age 22) | Child-completed questionnaire | YPB8023 |
|  | Frequency adult in family actually kicked, punched, hit respondent with something that could hurt respondent or physically attacked respondent in another way | Lifetime before age 11 | Categorical response:  Never/Rarely/Sometimes/Often/Very often | Yes = Rarely/  Sometimes/  Often/  Very often  No = Never | Before age of 11 | Child-completed questionnaire | YPB8006 |
|  | Frequency adult in family hit respondent so hard it left bruises or marks before age of 11 | Lifetime before age 11 | Categorical response:  Never/Rarely/Sometimes/Often/Very often | Yes = Rarely/  Sometimes/  Often/  Very often  No = Never | Before age of 11 | Child-completed questionnaire | YPB8007 |
|  | Frequency adult in family actually kicked, punched, hit respondent with something that could hurt respondent or physically attacked respondent in another way | Lifetime between ages 11 and 17 | Categorical response:  Never/Rarely/Sometimes/Often/Very often | Yes = Rarely/  Sometimes/  Often/  Very often  No = Never | Between ages of 11 and 17 | Child-completed questionnaire | YPB8056 |
|  | Frequency sibling actually kicked, punched, hit respondent with something that could hurt respondent or physically attacked respondent in another way between ages of 11 and 17 | Lifetime between ages 11 and 17 | Categorical response:  Never/Rarely/Sometimes/Often/Very often | Yes = Rarely/  Sometimes/  Often/  Very often  No = Never | Between ages of 11 and 17 | Child-completed questionnaire | YPB8067 |
|  | Frequency adult in family hit respondent so hard it left bruises or marks | Lifetime between ages 11 and 17 | Categorical response:  Never/Rarely/Sometimes/Often/Very often | Yes = Rarely/  Sometimes/  Often/  Very often  No = Never | Between ages of 11 and 17 | Child-completed questionnaire | YPB8057 |
|  | Amount YP has ever been aware of and affected by one 'parent' slapping, kicking, hitting or otherwise physically hurting the other | Lifetime | Categorical response:  Not at all/A little /A moderate amount/A lot | Yes = A little /A moderate amount/A lot  No = Not at all | Reported at age 21 |  | YPA5050 |
| Exposure to self-harm | Family member/close friend has self-harmed | Lifetime | Binary response:  Yes  No | N/A | 16 | Child-completed questionnaire | ccs6500 |
|  | Close friend has self-harmed | Lifetime | Binary response:  Yes  No | N/A | 16 | Child-completed questionnaire | ccs6520 |
| Body image | Degree to which respondent has been happy with the way their body looks | Past year | Categorical response:  Very unhappy/A little unhappy/Quite happy/Very happy | Happy = Quite happy/Very happy  Unhappy = Very unhappy/A little unhappy | 13 | Child-completed questionnaire | ccq440 |
| Drug and alcohol use | Number drinks YP usually has on a typical day when they have had a drink | N/A | Scale | Mean | 15.5 | Child-completed questionnaire | fh8545 |
|  | Drug use in past year | Past year | Binary: Yes/No | N/A | 15.5 | Child-completed questionnaire | fh8720-30 |
| Emotional-behavioural dysregulation (SDQ) | 5 subscales on prosocial behaviour, hyperactivity/inattention, emotional problems, conduct problems, peer problems | Current behaviour | Scale  0-10 | Mean | 13 | Child-based questionnaire (mother completed) | ta7025a, ta7025b, ta7025c, ta7025d  ta7025e |
| Callous unemotional traits | 6 questions on behaviour and emotions | Current behaviour | Categorical response:  Not at all/Rarely/Sometimes/Often/Always | Mean of total score:  Not at all= 0  Rarely = 1  Sometimes = 2  Often = 3  Always = 4 | 13 | Child-based questionnaire (mother completed) | tb3002, tb3004  tb3006, tb3009  tb3010, tb3012 |

**Table S2: Imputation model**

All participants with data on self-harm and violence outcome measures in Table S1 were included in analyses. Data on covariates were imputed. The imputation model included exposure, confounding and outcome variables in Table S1 plus auxiliary variables listed below.

| Variable name | Details | Type of variable |
| --- | --- | --- |
| bestgest | Gestation – length of pregnancy in weeks | Continuous |
| mz028b | Age of mother at delivery | Continuous |
| mz010a | Pregnancy size (singleton or multiple) | Binary |

**Table S3: Description of cohort: prevalence of exposures by dual harm status at ages 16 years (complete case)**

| **Characteristic/exposure** | **Neither self-harm nor violence** | | **Self-harm only** | | **Violence only** | | **Dual harm** | |
| --- | --- | --- | --- | --- | --- | --- | --- | --- |
| **Categorical measures** | **n** | **%** | **n** | **%** | **n** | **%** | **n** | **%** |
| Total | 2386 | 57.1 | 755 | 18.1 | 881 | 21.1 | 154 | 3.7 |
| Male | 1392 | 58.3 | 132 | 17.5 | 647 | 73.4 | 106 | 68.8 |
| Female | 994 | 41.7 | 623 | 82.5 | 234 | 26.6 | 48 | 31.2 |
| Low socioeconomic position | 449 | 19.4 | 178 | 24.9 | 254 | 31.1 | 42 | 28.8 |
| Depression (Short Mood and Feelings Questionnaire) score: Yes >=12) (age 13) | 133 | 5.8 | 116 | 20.6 | 71 | 8.9 | 35 | 24.6 |
| Victim of dating violence (age 13) | 110 | 8.3 | 45 | 11.5 | 117 | 18.7 | 17 | 13.9 |
| Hit by friends (age 12) | 189 | 8.0 | 79 | 13.1 | 153 | 18.3 | 34 | 22.5 |
| Hit by someone outside family before age 11 ^a^ | 11 | 0.7 | 8 | 1.7 | -- | -- | -- | -- |
| Hit by someone in family before age 11 (reported at age 22) | 67 | 4.0 | 46 | 9.8 | 49 | 14.4 | 14 | 14.4 |
| Hit with something by family before age 11 ^a^ | 61 | 3.7 | 49 | 10.4 | 50 | 14.7 | 9 | 9.5 |
| Hit by someone in family between age 11 and 17 ^a^ | 51 | 3.1 | 44 | 9.4 | 32 | 9.5 | 7 | 7.3 |
| Witnessed parental violence ever (reported at age 21) | 50 | 3.3 | 39 | 8.7 | -- | -- | -- | -- |
| Family member self-harmed (age 16) | 140 | 5.6 | 185 | 23.2 | 34 | 7.0 | 36 | 22.5 |
| Close friend self-harmed (age 16) | 779 | 31.3 | 609 | 76.6 | 136 | 27.9 | 127 | 78.9 |
| Happy with body image (age 13) | 1564 | 71.6 | 329 | 50.0 | 459 | 71.7 | 62 | 44.3 |
| Drug use in past year (age 15) | 37 | 1.6 | 26 | 5.3 | 75 | 9.4 | 23 | 15.1 |
| **Continuous measures** | **n** | **Mean** | **n** | **Mean** | **n** | **Mean** | **n** | **Mean** |
| Number of drinks when typically drinks alcohol (age 15) | 2381 | 1.5 | 506 | 2.1 | 854 | 2.5 | 154 | 2.9 |
| Score on 13-item beliefs about violence scale (age 13) | 2287 | 1.9 | 554 | 2.1 | 789 | 3.1 | 141 | 2.5 |
| Strengths and Difficulties Questionnaire (mean score) (age 13) | 2249 | 5.8 | 627 | 7.0 | 756 | 7.6 | 141 | 8.8 |
| Callous unemotional traits (age 13) | 2265 | 4.2 | 645 | 5.2 | 764 | 5.3 | 141 | 5.8 |

^a^ Reported at age 22

-- Cell count contains 5 or fewer participants so not reported

**Table S4: Description of cohort: prevalence of exposures by dual harm status at ages 22 years (complete case)**

| **Characteristic/exposure** | **Neither self-harm nor violence** | | **Self-harm only** | | **Violence only** | | **Dual harm** | |
| --- | --- | --- | --- | --- | --- | --- | --- | --- |
| **Categorical measures** | **n** | **%** | **n** | **%** | **n** | **%** | **n** | **%** |
| Total | 2046 | 43.3 | 1142 | 24.2 | 1217 | 25.8 | 321 | 6.8 |
| Female | 1196 | 58.5 | 895 | 78.4 | 364 | 29.9 | 219 | 68.2 |
| Male | 850 | 41.5 | 247 | 21.6 | 853 | 70.1 | 102 | 31.8 |
| Low socioeconomic position | 382 | 19.3 | 256 | 23.8 | 341 | 30.6 | 85 | 28.1 |
| Depression (Short Mood and Feelings Questionnaire) score: Yes >=12) (age 13) | 104 | 5.3 | 151 | 17.8 | 76 | 7.7 | 72 | 25.5 |
| Victim of dating violence (age 13) | 81 | 7.2 | 64 | 11.3 | 130 | 17.1 | 46 | 19.6 |
| Hit by friends (age 12) | 159 | 7.9 | 113 | 12.4 | 178 | 17.0 | 71 | 23.9 |
| Hit by someone outside family before age 11 ^a^ | 8 | 0.6 | 10 | 1.4 | -- | -- | -- | -- |
| Hit by someone in family before age 11 (reported at age 22) | 45 | 3.2 | 61 | 8.6 | 67 | 12.7 | 35 | 18.6 |
| Hit with something by family before age 11 ^a^ | 39 | 2.8 | 64 | 9.0 | 74 | 14.0 | 25 | 13.4 |
| Hit by someone in family between age 11 and 17 ^a^ | 30 | 2.2 | 57 | 8.0 | 56 | 10.7 | 23 | 12.4 |
| Witnessed parental violence ever (reported at age 21) | 34 | 2.7 | 44 | 6.9 | 34 | 8.2 | 23 | 12.2 |
| Family member self-harmed (age 16) | 107 | 5.0 | 186 | 19.5 | 51 | 7.5 | 67 | 23.4 |
| Close friend self-harmed (age 16) | 644 | 30.2 | 650 | 68.3 | 191 | 28.2 | 212 | 73.6 |
| Happy with body image (age 13) | 1353 | 72.4 | 488 | 53.0 | 622 | 72.8 | 134 | 48.7 |
| Drug use in past year (age 15) | 29 | 1.5 | 37 | 5.0 | 68 | 7.4 | 44 | 15.8 |
| **Continuous measures** | **n** | **Mean** | **n** | **Mean** | **n** | **Mean** | **n** | **Mean** |
| Number of drinks when typically drinks alcohol (age 15) | 2035 | 1.4 | 766 | 2.0 | 787 | 2.4 | 291 | 2.8 |
| Score on 13-item beliefs about violence scale (age 13) | 1956 | 1.9 | 843 | 1.9 | 971 | 2.9 | 280 | 2.8 |
| Strengths and Difficulties Questionnaire (mean score) (age 13) | 1924 | 5.7 | 918 | 6.8 | 987 | 7.4 | 283 | 8.6 |
| Callous unemotional traits (age 13) | 1932 | 4.1 | 948 | 5.1 | 996 | 5.2 | 289 | 5.9 |

^a^ Reported at age 22

**Table S5: Unadjusted relative risk ratios (RRR) for the association between exposures and dual harm status at age 16 and 22 years (pooled proportions from imputed data)**

|  | **Age 16 years** | | | | **Age 22 years** | | | |
| --- | --- | --- | --- | --- | --- | --- | --- | --- |
|  | **Neither self-harm or violence (N= 2,386)** | **Self-harm only (N= 755)** | **Violence only (N= 881)** | **Dual harm (N= 154)** | **Neither self-harm or violence (N= 2,046)** | **Self-harm only (N= 1,142)** | **Violence only (N=1,217)** | **Dual harm (N=321)** |
| **Characteristic/ exposure** | **RRR** | **RRR (95% CI)** | **RRR (95% CI)** | **RRR (95% CI)** | **RRR** | **RRR (95% CI)** | **RRR (95% CI)** | **RRR (95% CI)** |
| Mean depression score (overall 0-26) (age 13) | 1 (ref) | 1.16 (1.14-1.18) | 1.07 (1.04-1.09) | 1.17 (1.14-1.21) | 1 (ref) | 1.15 (1.13-1.17) | 1.07 (1.05-1.09) | 1.19 (1.16-1.22) |
| Depression (Short Mood and Feelings Questionnaire) score: Yes >=12) (age 13) | 1 (ref) | 3.81 (2.91-4.91) | 1.61 (1.19-2.20) | 5.07 (3.31-7.77) | 1 (ref) | 3.22 (2.47-4.20) | 1.49 (1.10-2.04) | 5.28 (3.77-7.40) |
| Mean attitude to violence score (age 13) | 1 (ref) | 1.10 (1.04-1.16) | 1.44 (1.37-1.51) | 1.24 (1.13-1.37) | 1 (ref) | 1.05 (0.99-1.10) | 1.37 (1.31-1.44) | 1.35 (1.26-1.45) |
| Victim of dating violence (age 13) | 1 (ref) | 1.59 (1.15-2.19) | 2.36 (1.79-3.10) | 1.92 (1.14-3.23) | 1 (ref) | 1.55 (1.15-2.07) | 2.23 (1.71-2.92) | 2.73 (1.88-3.96) |
| Hit by friends (age 12) | 1 (ref) | 1.75 (1.32-2.31) | 2.55 (2.02-3.21) | 3.47 (2.30-5.24) | 1 (ref) | 1.65 (1.28-2.14) | 2.35 (1.88-2.95) | 3.66 (2.66-5.03) |
| Hit by someone outside family before age 11 (reported at age 22) | 1 (ref) | 2.22 (0.97-5.08) | 3.06 (1.44-6.50) | 3.96 (1.36-11.56) | 1 (ref) | 2.03 (0.96-4.32) | 3.16 (1.58-6.33) | 3.56 (1.37-9.25) |
| Hit by someone in family before age 11 (reported at age 22) | 1 (ref) | 2.52 (1.76-3.62) | 2.72 (1.96-3.77) | 3.73 (2.09-6.67) | 1 (ref) | 2.51 (1.71-3.68) | 2.93 (2.06-4.16) | 5.15 (3.32-7.98) |
| Hit with something by family before age 11 (reported at age 22) | 1 (ref) | 2.66 (1.82-3.89) | 3.04 (2.14-4.32) | 2.84 (1.48-5.48) | 1 (ref) | 2.62 (1.77-3.87) | 3.40 (2.38-4.85) | 4.34 (2.71-6.95) |
| Hit by someone in family between age 11 and 17 (reported at age 22) | 1 (ref) | 3.04 (2.05-4.50) | 2.69 (1.83-3.96) | 2.68 (1.20-6.00) | 1 (ref) | 2.94 (1.94-4.44) | 3.30 (2.22-4.90) | 5.02 (3.03-8.31) |
| Witnessed parental violence ever (age 21) | 1 (ref) | 2.43 (1.63-3.64) | 2.18 (1.43-3.31) | 1.94 (0.77-4.88) | 1 (ref) | 2.26 (1.45-3.51) | 2.35 (1.55-3.57) | 4.02 (2.44-6.61) |
| Family member self-harmed (age 16) | 1 (ref) | 5.10 (3.99-6.51) | 1.60 (1.13-2.26) | 4.64 (3.04-7.09) | 1 (ref) | 4.20 (3.25-5.44) | 1.79 (1.30-2.49) | 5.23 (3.70-7.38) |
| Close friend self-harmed (age 16) | 1 (ref) | 7.58 (6.26-9.19) | 1.05 (0.87-1.27) | 8.21 (5.54-12.19) | 1 (ref) | 4.39 (3.74-5.16) | 1.10 (0.92-1.30) | 5.51 (4.19-7.25) |
| Happy with body image (age 13) | 1 (ref) | 0.42 (0.35-0.50) | 0.99 (0.82-1.19) | 0.33 (0.23-0.46) | 1 (ref) | 0.47 (0.39-0.55) | 0.97 (0.81-1.16) | 0.36 (0.28-0.48) |
| Mean number of drinks when typically drinks alcohol (age 15) | 1 (ref) | 1.18 (1.13-1.24) | 1.29 (1.24-1.34) | 1.41 (1.31-1.52) | 1 (ref) | 1.17 (1.12-1.22) | 1.27 (1.22-1.32) | 1.40 (1.31-1.48) |
| Drug use in past year (age 15) | 1 (ref) | 3.61 (2.21-5.88) | 5.53 (3.67-8.33) | 10.53 (6.06-18.30) | 1 (ref) | 3.35 (2.07-5.42) | 4.62 (3.00-7.13) | 10.20 (6.21-16.74) |
| Strengths and Difficulties Questionnaire (mean score) (age 13) | 1 (ref) | 1.06 (1.04-1.08) | 1.08 (1.06-1.10) | 1.13 (1.09-1.16) | 1 (ref) | 1.06 (1.04-1.08) | 1.08 (1.06-1.10) | 1.13 (1.10-1.15) |
| Callous unemotional traits (age 13) | 1 (ref) | 1.12 (1.09-1.15) | 1.12 (1.09-1.15) | 1.18 (1.13-1.24) | 1 (ref) | 1.11 (1.08-1.14) | 1.13 (1.10-1.16) | 1.19 (1.15-1.24) |
